# Antifungal Properties of Hydrazine-Based Compounds against *Candida albicans*

**DOI:** 10.3390/antibiotics12061043

**Published:** 2023-06-12

**Authors:** Louis Camaioni, Dylan Lambert, Boualem Sendid, Muriel Billamboz, Samir Jawhara

**Affiliations:** 1 CNRS, UMR 8576-UGSF—Unité de Glycobiologie Structurale et Fonctionnelle, INSERM U1285, F-59000 Lille, France; louis.camaioni@gmail.com (L.C.); dylan.lambert2.etu@univ-lille.fr (D.L.); boualem.sendid@univ-lille.fr (B.S.); 2 Medicine Faculty, University of Lille, F-59000 Lille, France; 3 CHU Lille, Service de Parasitologie Mycologie, Pôle de Biologie Pathologie Génétique, F-59000 Lille, France; 4 INSERM, CHU Lille, Institut Pasteur Lille, U1167-RID-AGE—Facteurs de Risque et Déterminants Moléculaires des Maladies Liées au Vieillissement, University of Lille, F-59000 Lille, France; muriel.billamboz@junia.com; 5 JUNIA, Health and Environment, Laboratory of Sustainable Chemistry and Health, F-59000 Lille, France

**Keywords:** *Candida albicans*, hydrazine, biofilm, fungal resistance, *Caenorhabditis elegans* infection model

## Abstract

*Candida albicans*, an opportunistic yeast, is the most common cause of fungal infection. In the past decade, there has been an increase in *C. albicans* resistance to existing antifungal drugs, which has necessitated the development of new antifungal agents. In the present study, screening 60 compounds from the JUNIA chemical library enabled us to explore an additional 11 hybrid compounds that contain pyrrolidinone rings and hydrazine moieties for their potential antifungal activities. This chemical series was identified with fair to excellent antifungal activities. Among this series, three molecules (Hyd.H, Hyd.OCH_3_, and Hyd.Cl) significantly reduced *C. albicans* viability, with rapid fungicidal activity. In addition, these three compounds exhibited significant antifungal activity against clinically isolated fluconazole- or caspofungin-resistant *C. albicans* strains. Hyd.H, Hyd.OCH_3_, and Hyd.Cl did not show any cytotoxicity against human cancer cell lines up to a concentration of 50 µg/mL and decreased *Candida* biofilm formation, with a significant reduction of 60% biofilm formation with Hyd.OCH_3_. In an infection model of *Caenorhabditis elegans* with *C. albicans*, hydrazine-based compounds significantly reduced nematode mortality. Overall, fungicidal activity was observed for Hyd.H, Hyd.OCH_3_, and Hyd.Cl against *C. albicans*, and these compounds protected *C. elegans* from *C. albicans* infection.

## 1. Introduction

Opportunistic fungal infections represent a serious cause of morbidity and mortality in immunocompromised or hospitalized patients with serious underlying diseases [1,2]. The opportunistic fungal pathogen *Candida albicans* colonizes the oropharyngeal cavity, gastrointestinal mucosa, and vaginal tract [1,3]. *C. albicans* overgrowth in these niches can cause superficial mucosal infections and life-threatening systemic diseases, making *C. albicans* the main opportunistic fungal pathogen in humans [1,3]. *C. albicans* is also the most frequently identified *Candida* species causing nosocomial disease in hospital settings. Resistance to antifungal drugs is an emerging worldwide health problem and creates difficulties in selecting the correct antifungal therapy [4]. *C. albicans* has acquired resistance to many antifungal drugs, including azoles and echinocandins. This increase in *C. albicans* resistance is due to prolonged exposure to current antifungal drugs and, in particular, the excessive use of azoles and echinocandins in patients at high risk of invasive candidiasis [5,6].

An important virulence factor of *C. albicans* is its capacity to develop biofilms, densely packed communities of cells adhering to a surface. *C. albicans* biofilms containing hyphae, pseudohyphae, and yeast cells are resistant to conventional antifungal therapeutics and the immune response [7]. Most antifungal drugs are unable to penetrate biofilms. There is the potential for candidemia relapse, which leads to high rates of mortality and morbidity [8]. To investigate the impact of new antifungal compounds on fungal infection and pathogenesis progression, the nematode *Caenorhabditis elegans* is a well-established model to study *C. albicans* infection and the host’s innate immune response. A yeast form of *C. albicans* is ingested into the digestive tract of *C. elegans* [9,10], and the yeast form then switches to the hyphal form in the liquid medium, causing tissue damage, aggressive infection, and death of the nematode [9]. In a *C. elegans* infection model, two compounds, 1-(4-chlorophenyl)-4-((4-chlorophenyl)amino)-3,6-dimethylpyridin-2(*1H*)-one and (Z)-*N*-(2-(4,6-dimethoxy-1,3,5-triazin-2-yl)vinyl)-4-methoxyaniline, were recently shown to have antifungal activity against *C. albicans* [11].

In the present study, 60 potential antifungal compounds belonging to a dozen different chemical series were selected from the JUNIA chemical library. They were evaluated for their antifungal activity against *C. albicans*. Notably, a wide variety of synthetic compounds were analyzed, including carbazate or saccharine derivatives, triazenes, as well as bio-sourced compounds derived from menthol, eugenol, kojic acid, or sesamol. After conducting this preliminary screening as well as measuring their MICs against the *C. albicans* wild-type strain, it was found that hybrid molecules containing pyrrolidinone rings coupled with hydrazine moiety molecules provided fair to excellent antifungal effects against *C. albicans*. Next, a panel of 11 molecules from this series was submitted for biological evaluation to assess their antifungal activities. Three compounds (Hyd.H, Hyd.OCH_3_, and Hyd.Cl) were identified from this panel that showed potential antifungal activity against *C. albicans*. These three compounds were then evaluated for their antifungal activities against *Candida* viability, *Candida* biofilms, and clinical antifungal-resistant *Candida* strains. In a *C. elegans* infection model, these three antifungal compounds affected *C. albicans* virulence and protected *C. elegans* from mortality due to *C. albicans* infection.

## 2. Results

**Figure 1:**
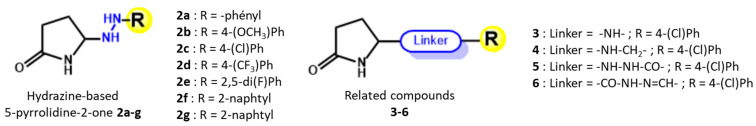
Structure of the compounds used in the current study.

The selected series of hydrazine-based 5-pyrrolidine-2-one compounds were evaluated and compared with related chemical substances (Figure 1) and caspofungin as a reference.

### 2.1. Antifungal Activities of Hydrazine-Based Compounds against C. albicans Wild-Type Strain

After screening selection, a panel of 11 molecules, composed of a pyrrolidinone ring coupled with a nitrogen donating group, was selected and evaluated against the *C. albicans* wild-type strain (Figure 1). The results are summarized in Table 1. Seven compounds are based on hydrazine moieties (**2a**–**g**). Related molecules (**3**–**6**), incorporating other linkers, were integrated into the test panel in order to decipher the role of the hydrazine linker. Amine (**3** and **4**), hydrazide (**5**), and hydrazone (**6**) linkers were chosen.

Following this panel, compounds **2a** (Hyd.H), **2b** (Hyd.OCH_3_), and **2c** (Hyd.Cl) showed excellent activity against *C. albicans*, whereas hydrazine-based lactams (**2d**–**g**) displayed lower antifungal activity, comparable with those of compounds **3**–**6**. Compound **2a** exhibited potential activity against *C. albicans*, with MIC = 9.6 µg/mL (Table 1, entry 1). The addition of an electro-donating methoxy group in the para-position of the aromatic moiety, leading to compound **2b**, decreased the antifungal activity slightly (Table 1, entry 2). The integration of a para-chlorine atom on the aromatic moiety led to compound **2c**, with increased activity against *C. albicans* (MIC = 5.6 µg/mL) (Table 1, entry 3). Adding a strong electro-withdrawing group (-CF_3_), yielding **2d**, decreased the activity, suggesting that the electronic ability of the substituents is crucial against *C. albicans*. The replacement of the phenyl moiety with a 2-pyridine in compound **2f** led to a significant loss of activity against *C. albicans* (Table 1, entry 6). Moreover, steric hindrance seemed to be important since compounds **2e** and **2g**, bearing a difluorinated aromatic and a naphthyl group, respectively, showed very low potency (Table 1, entries 5 and 7). From this first screening, compound **2c**, bearing a para-chlorophenyl moiety, was selected as the most interesting antifungal compound against *C. albicans* for the evaluation of linker replacement.

To assess the role of the hydrazine linker, related structures (**3**–**6**) were designed and evaluated (Figure 1). Replacing the 4-chlorohydrazine moiety with a 4-chloroaniline in **3** or a 4-chlorobenzylamine in **4** led to a strong decrease in activity, with MICs >100 µg/mL (Table 1, entries 8 and 9). This modification proved the importance of the second -NH- group to achieve high antifungal activity against *C. albicans*. This could be due to its proper hydrogen bonding capacity. Changing the hydrazine with a carbazide link in compound **5** also caused a dramatic loss of potency (Table 1, entry 10), proving that the addition of a carbonyl group in the link should be avoided. The integration of an acylhydrazone link led to compound **6**, which did not show any antifungal potential. Again, increasing the length of the link by introducing a carbonyl group and losing the second -NH- group was deleterious for antifungal activity.

Combining these results, compounds **2a** (Hyd.H), **2b** (Hyd.OCH_3_), and **2c** (Hyd.Cl) were selected for further experiments.

### 2.2. Antifungal Effects of Hydrazine-Based Compounds on Drug-Resistant Clinical Isolates of C. albicans

**Figure 2:**
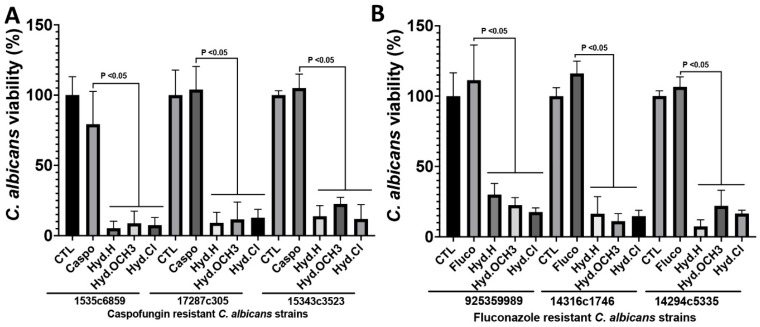
Impact of hydrazine compounds on the viability of *C. albicans* clinical isolates resistant to caspofungin and fluconazole. (**A**) Caspofungin resistant *C. albicans* strains. (**B**) Fluconazole resistant *C. albicans* strains. *C. albicans* clinical isolates were treated with hydrazine compounds at 2× their MICs. *C. albicans* viability was determined using the Alamar Blue reagent after 24 h challenge. CTL: *C. albicans* clinical strain without antifungal treatment; Caspo: *C. albicans* cells challenged with caspofungin; Fluco: *C. albicans* cells challenged with fluconazole.

To determine whether these compounds had any antifungal activity against drug-resistant clinical isolates of *C. albicans*, fluconazole- or caspofungin-resistant strains isolated from patients (Table 2) were challenged with these compounds. The viability of *C. albicans* isolates resistant to either fluconazole or caspofungin was significantly reduced after treatment with either Hyd.H, Hyd.OCH_3_, or Hyd.Cl at 2× their MICs (Figure 2). These data show that the selected compounds are broad-spectrum agents able to inhibit drug-resistant strains, which is currently of particular concern.

### 2.3. C. albicans Biofilms

**Figure 3:**
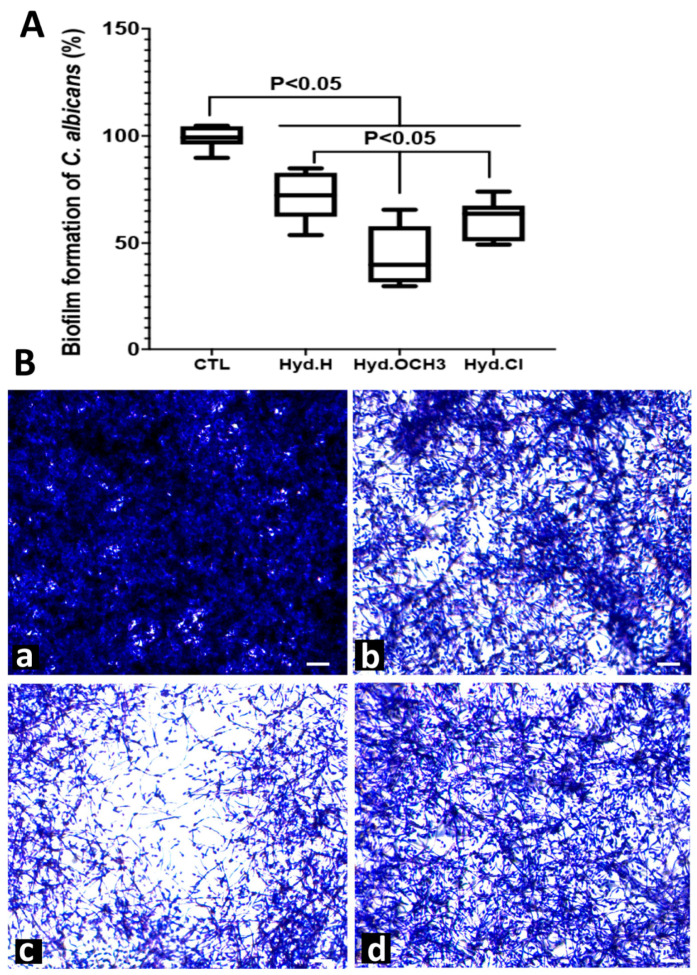
Effect of hydrazine compounds on *C. albicans* biofilm formation: (**A**) *C. albicans* cells formed a dense biofilm after 48 h. Hydrazine compounds at 1× their MICs were added to the *C. albicans* biofilms for 24 h. CTL: *C. albicans* alone without any antifungal treatment; (**B**) *C. albicans* biofilm challenged with (**a**) CTL: *C. albicans* alone without any antifungal treatment, (**b**) Hyd.H, (**c**) Hyd.OCH_3_, and (**d**) Hyd.Cl. Scale bars represent 10 µm.

To assess whether hydrazine-based compounds affected *C. albicans* biofilm formation, which is implicated in resistance to several antifungal agents, including azoles and echinocandins, *C. albicans* biofilms were challenged with Hyd.H, Hyd.OCH_3_, or Hyd.Cl at 1× their MICs (9.6 µg/mL, 11.1 µg/mL, and 5.6 µg/mL, respectively) (Figure 3). The three antifungal agents inhibited *C. albicans* biofilm formation. Hyd.OCH_3_ significantly reduced biofilm formation, by 60%. Furthermore, HyD.OCH_3_ showed significantly higher biofilm inhibition when compared to Hyd.H and Hyd.Cl (*p* < 0.05). No significant difference between Hyd.H and Hyd.Cl was observed. Microscopic examination showed that the biofilm matrix was dense and highly compacted when *C. albicans* was challenged with phosphate-buffered saline (PBS) as a control (CTL), whereas when challenged with antifungal compounds, the biofilm appeared to dissolve, and the *C. albicans* cells detached from the biofilm matrix (Figure 3).

### 2.4. Analysis of Human Cancer Cell Line Viability after Treatment with Different Concentrations of Hydrazine-Based Compounds

**Figure 4:**
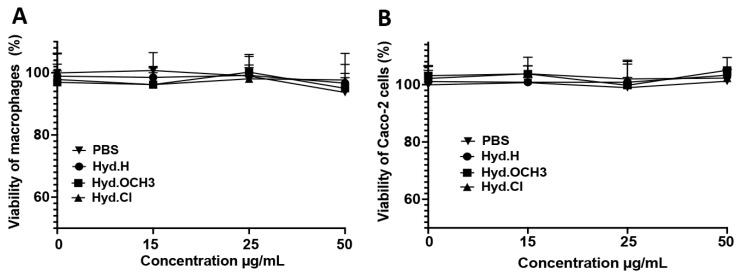
Evaluation of the cytotoxicity of the three compounds against human cancer cell lines using the MTT assay. Analysis of the cytotoxicity of these three compounds against macrophages (**A**) and Caco-2 (**B**) cells.

According to previous assays, the three selected compounds Hyd.H, Hyd.OCH_3,_ and Hyd.Cl displayed potent antifungal and antibiofilm capacities. However, before the evaluation of their in vivo activity in a *C. elegans* model, an evaluation of their cytotoxicity profile was performed. Indeed, in the search for new active agents, any cytotoxic compounds should be detected and discarded. In this context, increasing dilutions of Hyd.H, Hyd.OCH_3,_ and Hyd.Cl higher than their MICs (9.6 µg/mL, 11.1 µg/mL, and 5.6 µg/mL, respectively) were incubated with human cancer cell lines (THP-1 macrophages and intestinal Caco-2 cells), and the percent cell viability was determined using an MTT assay. MTT assay is a standardized reference assay to determine the potential of a compound to proceed to in vivo evaluation. The cytotoxicity of these three compounds against macrophages and intestinal Caco-2 cells was measured (Figure 4). In both cell lines, the three selected molecules did not show any cytotoxicity against human cancer cells up to a concentration of 50 µg/mL (Figure 4). According to these data, the three compounds could proceed to the next in vivo step.

### 2.5. Hydrazine-Based Compounds Confer Increased Survival of C. elegans against C. albicans Infection

**Figure 5:**
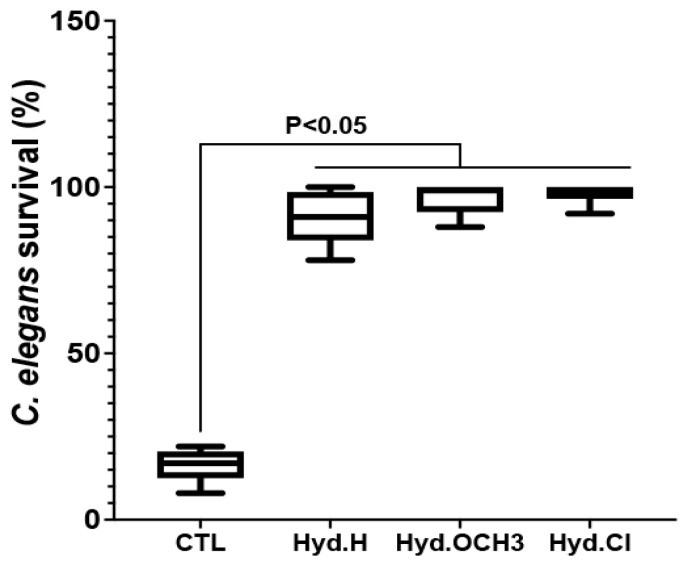
Effects of hydrazine compounds on *C. albicans* pathogenesis in a *C. elegans* infection model. Nematodes infected with *C. albicans* were assessed for survival daily for 4 days, and the percentage of worm survival was evaluated on day 4. Nematodes infected with *C. albicans* were treated with Hyd.H, Hyd.OCH_3_, or Hyd.Cl at their MICs (9.6 µg/mL, 11.1 µg/mL, and 5.6 µg/mL, respectively). A platinum wire pick was used to determine the death of the nematodes if they failed to respond to contact. CTL: nematodes infected with *C. albicans* without antifungal treatment; Hyd.H, Hyd.OCH_3_, and Hyd.Cl: nematodes infected with *C. albicans* and treated with hydrazine compounds.

The impact of hydrazine-based compounds on *C. albicans* wild-type strain pathogenesis in vivo was explored using a *C. elegans* infection model (Figure 5). Nematodes infected with *C. albicans* were treated with Hyd.H, Hyd.OCH_3_, or Hyd.Cl at their MICs. The survival of *C. elegans* was examined daily using microscopic observation. As a control, infection with *C. albicans* caused 85% nematode mortality by day 4. The treatment of worms with hydrazine-based compounds showed a higher rate of *C. elegans* survival when compared to untreated worms (around 15% survival in the control compared to 90–99% survival after treatment). The percent survival of nematodes treated with Hyd.OCH_3_ or Hyd.Cl was slightly higher than that of *C. elegans* treated with Hyd.H (Figure 5).

## 3. Discussion

The increasing frequency of drug resistance among fungal pathogens poses a serious threat to public health and healthcare systems worldwide [14]. It is urgent to develop new antifungals that are effective against clinically relevant fungal pathogens as well as in defeating drug resistance in fungi [15]. In the present study, our initial screening of 60 compounds from the JUNIA chemical library allowed us to explore a further panel of 11 hybrid compounds with pyrrolidinone rings and hydrazine moieties for their antifungal properties. From this panel, three compounds (Hyd.H, Hyd.OCH_3_, and Hyd.Cl) showed antifungal activity. These three hydrazine-based drug candidates showed antifungal activity against the *C. albicans* wild-type strain and were also effective against drug-resistant clinical *C. albicans* isolates. Different studies have emphasized the role of hydrazine derivatives as promising antifungal agents [16,17,18]. Zhang et al. showed that a series of citral-thiazolyl hydrazine derivatives caused an obvious malformation of mycelium and increased the permeability of cell membranes, demonstrating that these derivatives possess remarkable antifungal activity against phytopathogenic fungi [17]. In line with this study, the hydrazine compound 4-phenyl-1, 3-thiazol-2-yl induced oxidative damage in *C. albicans* and exhibited fungicidal activity while having low toxicity to human cancer cells [16].

**Figure 6:**
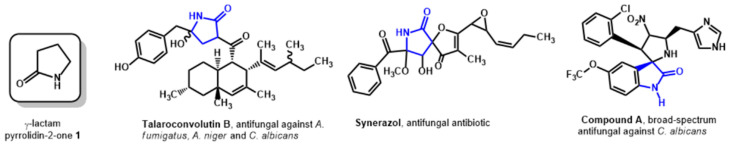
Compounds with reported antifungal activity against *C. albicans* bearing a γ-lactam ring.

A recent study showed that hydrazine and acyl hydrazone derivatives of 5-pyrrolidine-2-one were effective antifungal agents against a series of 12 fungal strains as well as 3 non-albicans *Candida* species [18]. Several hydrazine derivatives have also been found to have good antifungal properties against *Zymoseptoria tritici*, the main pathogen of wheat plants [19]. Pyrrolidine-2-one, or γ-lactam 1, is a heterocyclic moiety widespread in many natural compounds. As an illustration, talaroconvolutin B, extracted from *Talaromyces convolutes*, is highly active against *Aspergillus fumigatus*, *A. niger*, and *C. albicans* [20]. Synerazol, an antibiotic as well as an antifungal, isolated from cultures of *A. fumigatus*, also exhibited high potential against *C. albicans* [21]. Accordingly, structural features derived from natural γ-lactams are a significant inspiration to design innovative biologically active molecules. As a result of this new design, spirooxindole pyrrolidine tethered indole–imidazole hybrid heterocycle compound A (Figure 6) showed broad-spectrum antifungal activity against *Candida* species. 

In the present study, compound **2a** showed antifungal activity against *C. albicans*, while the addition of an electro-donating methoxy group in the para-position of the aromatic moiety affected its antifungal activity [18]. Furthermore, the antifungal activity of Hyd.CF_3_ (compound **2d**) was found to be lower than that of Hyd.H, Hyd.OCH_3_, and Hyd.Cl, indicating that the electronic ability of substituents plays a significant role in the antifungal activity of these compounds against *C. albicans*. However, the integration of an electro-withdrawing group (-CF_3_) was crucial to enhance the antifungal efficacy of this compound against *Z. tritici* [18]. Damiens et al. demonstrated that the integration of an acylhydrazone link (compound **6**) resulted in antifungal activity against *Z. tritici*. However, the same compound did not show any antifungal potential against *C. albicans*, which implies that *C. albicans* behaves in a different way from *Z. tritici* towards this series of compounds, indicating that they have a different biological target [19].

The challenge of *C. albicans* with hydrazine compounds resulted in a significant reduction in *C. albicans* viability as well as a fast killing rate. These data suggest that these compounds have fungicidal activity against *C. albicans*. In addition, these compounds also reduced the viability of *C. albicans* clinical isolates that were resistant to either fluconazole or caspofungin.

*C. albicans* biofilms are intrinsically resistant to conventional antifungal drugs, making biofilm-associated infections difficult to combat [7]. In the present study, hydrazine compounds decreased biofilm formation by *C. albicans*, particularly Hyd.OCH_3_. These three compounds did not have any cytotoxicity towards human cancer cell lines, even at 5x their MICs. The effect of these compounds on the elimination of *C. albicans* infection was assessed using the nematode *C. elegans.* In recent years, this nematode has been widely used as a model for investigating *C. albicans* pathogenesis [10]. The present study showed that hydrazine compounds protected *C. elegans* from *C. albicans* infection, and the compounds Hyd.OCH_3_ and Hyd.Cl were the most effective against *C. albicans* infection.

The results of this study are in line with our recent finding that both TRI (for triazine derivatives) and PYR (for pyridinone) reduced the mortality rate of nematodes infected with *C. albicans* [11]. This suggests that, in addition to the in vitro screening of antifungal compounds, the *C. elegans* infection model is an important step forward in the identification of active antifungal compounds [11]. Notably, the structure of neither Hyd.OCH_3_ nor Hyd.Cl has been described in the literature. These two compounds were synthesized for this study and were shown to have antifungal properties against *C. albicans*.

In conclusion, hydrazine-based compounds showed fungicidal activity with a fast killing rate of *C. albicans* and were highly effective against clinical isolates of *C. albicans* resistant to antifungal drugs. In addition, they also decreased biofilm formation and did not exhibit any cytotoxicity against macrophages and intestinal Caco-2 cells. These compounds could protect *C. elegans* against *C. albicans* infection. Overall, these data suggest that hydrazine-based compounds may be the lead compounds for the development of novel antifungal drugs.

## 4. Materials and Methods

### 4.1. Materials

For the chemical compounds, all tested molecules were designed, synthesized, and provided by JUNIA-Hautes Etudes d’Ingénieur (HEI), Lille, France. They were fully characterized and indexed at the Laboratory of Sustainable Chemistry and Health from JUNIA. They were employed at their MICs, respectively, and diluted in PBS (Fisher Scientific, Illkirch, France) during the various in vitro and in vivo experiments. Commercially available caspofungin (Merck, Semoy, France) and fluconazole (Fresenius, Sèvres, France) were used as positive controls. For cell line cultures, THP-1 (ATCC TIB-202) and Caco-2 cells (ATCC HTB-37) were cultured in an RPMI-1640 medium (Fisher Scientific, Illkirch, France) supplemented with fetal calf serum (10% *v*/*v*; Sigma-Aldrich, St. Quentin Fallavier, France) and penicillin–streptomycin (1% *v*/*v*; Fisher Scientific, Illkirch, France). For fungal and bacterial cultures, *C. albicans* strains were cultured in Sabouraud dextrose broth (Sigma-Aldrich, St. Quentin Fallavier, France) at 37 °C for 24 h. The *Escherichia coli* strain OP50 was cultured in Luria broth (Sigma-Aldrich, St. Quentin Fallavier, France) at 37 °C for 12 h.

### 4.2. C. albicans Strains

*C. albicans* SC5314 was the wild-type strain (ATCC MYA-2876) used in this study (Table 2) [12]. *C. albicans* cells were cultivated on Sabouraud dextrose agar (SDA) for 24 h at 37 °C [22]. For the preparation of *C. albicans* suspensions, *C. albicans* cells were cultured in Sabouraud dextrose broth (Sigma-Aldrich, France) for 24 h at 37 °C in a rotary shaker. After different washes with PBS, *C. albicans* yeast cells were then centrifuged at 2500 rpm for 5 min and resuspended in PBS. For the clinical *C. albicans* strains, six strains of *C. albicans* isolated from patients that were resistant to either fluconazole or caspofungin were cultured in SDA for 24–48 h. Their MICs were determined using the standard culture microdilution method from the Clinical and Laboratory Standard Institute, as described previously by Pfaller et al. [23] (Table 2). For the identification of these clinical isolates, 1.5 μL of matrix solution (α-cyano-4-hydroxycinnamic acid; Bruker Daltonics, Billerica, MA, USA) was added to each *C. albicans* clinical isolate colony and mixed with 50% acetonitrile, 47.5% water, and 2.5% trifluoroacetic acid. The identification of these strains was performed using MALDI-TOF MS (Microflex, Bruker Daltonics) [24].

### 4.3. Fungal Viability Assays

The hydrazine-based compounds were developed, synthesized, and supplied by JUNIA. Multiple small batch aliquots were generated for each compound and kept in frozen storage at −20 °C. For each experiment, fresh aliquots were diluted in PBS and adjusted to the appropriate concentration for the experiment. To determine the MIC of these hydrazine-based compounds, and thus be able to characterize them, Alamar Blue reagent (Thermo Fisher Scientific, Illkirch-Graffenstaden, France) was employed in the assay [25]. As positive controls, caspofungin and fluconazole were also employed at their MICs. Alamar Blue is metabolized by the yeast, making it possible to determine the fungal cell activity and MIC. Alamar Blue (10 μL) was added to each well containing 5 × 10^3^ yeasts in 90 μL of RPMI medium. As for the antifungals, the hydrazine-based compounds were used at different concentrations (ranging from 5 × 10^−3^ M to 5 × 10^−6^ M in the well). The MIC was determined for each molecule as the concentration that resulted in 99% growth inhibition of the yeast. Measurements were performed at T0 and T24 by measuring the absorbance at 600 nm in a FLUOstar OMEGA spectrophotometer. In addition, 5 × 10^3^ *C. albicans* cells were incubated for 1 h with the antifungal compounds at 2× their MICs, and a 100 µL aliquot of each dilution was plated on SDA and incubated for 48 h to determine the viability.

### 4.4. C. albicans Biofilm Formation

*C. albicans* cell suspensions were adjusted to 5 × 10^3^ yeast cells in 200 µL of RPMI medium with 10% fetal calf serum. This suspension was added to individual wells in a polystyrene plate (Greiner Bio-one, Kremsmünster, Austria). The plates were then incubated for 48 h at 37 °C. Different washes with PBS were performed to remove non-adherent yeast cells. Hyd.H, Hyd.OCH_3_, and Hyd.Cl compounds at 1× their MICs (9.6 µg/mL, 11.1 µg/mL, and 5.6 µg/mL, respectively) were then added to the plate for 24 h. After 24 h incubation, the wells were washed with PBS and air-dried at 37 °C. Biofilms were stained with 0.4% crystal violet solution (Fluka) for 20 min. After multiple washes with PBS, 200 µL of ethanol was added to each well. The absorbance of the destaining solution that reflects the number of viable *C. albicans* cells was measured at 550 nm using a spectrophotometer (FLUOstar; BMG Labtech, Champigny sur Marne, France). All data are represented as the average of six replicates of two independent experiments.

### 4.5. Cytotoxicity Analysis

THP-1 cells (human leukemia monocytic cell line) were maintained in an RPMI-1640 medium supplemented with fetal calf serum (10% *v*/*v*) and penicillin–streptomycin (1% *v*/*v*). THP-1 cells were differentiated into macrophages by adding phorbol-12-myristate-13-acetate (Sigma-Aldrich, Saint-Quentin-Fallavier, France) at a concentration of 200 ng/mL for 72 h. Caco-2 cells (cell line derived from a human colorectal adenocarcinoma), and macrophages were treated with different concentrations of hydrazine-based compounds (25, 50, and 100 µg/mL) for 24 h at 37 °C at a concentration of 2 × 10^5^ cells/well in a 96-well transparent plate (Greiner Bio-one; 655101). After incubation, 10 µL of 3-(4,5-dimethylthiazol-2-yl)-2,5-diphenyltetrazolium bromide (MTT) reagent was added to each well [26]. The plate was kept at 37 °C in 5% CO_2_ for 4 h to assess the metabolic activity of the cells and to determine the viability of the Caco-2 cells and THP-1 cells. MTT detergent (100 μL) was added, and the absorbance was read at 570 nm using a spectrophotometer. The percent toxicity with respect to the negative control (cells + PBS) was calculated and plotted against the concentration of the compounds.

### 4.6. C. elegans Survival Assay

*C. albicans* strain SC5314 was grown in Sabouraud dextrose broth at 37 °C for 24 h. *C. albicans* lawns were established by spreading 10 µL of *C. albicans* culture on brain heart infusion plates containing amikacin (45 µg/mL). These plates were then incubated at 37 °C for 24 h. Wild-type N2 *C. elegans* was grown on a nematode growth medium seeded with *E. coli* strain OP50 at 20 °C. Worm populations were synchronized and incubated at 20 °C [27]. About 100 nematodes were picked for each experiment. These worms were washed at different times with an M9 buffer containing 90 µg/mL amikacin to remove *E. coli* and then added to the *C. albicans* lawns. The plates were incubated at room temperature for 6 h. The worms were then carefully washed multiple times with the M9 buffer to eliminate *C. albicans* cells from their cuticles. About 70–80 nematodes infected with *C. albicans* were then added to the wells in a 6-well microtiter dish that contained 2 mL of 80% liquid M9 buffer, 20% BHI, 10 µg/mL cholesterol in ethanol, and 90 µg/mL amikacin. Hyd.H, Hyd.OCH_3_, and Hyd.Cl at their MICs (9.6 µg/mL, 11.1 µg/mL, and 5.6 µg/mL, respectively) were then added to each well. The plates were incubated at room temperature for 12 h. Worms were examined daily for survival for 4 days and considered to be dead if they did not move in response to mechanical stimulation with a pick.

### 4.7. Statistical Analysis

The Mann–Whitney U test was used to determine the differences between the groups, and the data were statistically significant when the *p* value was *p* < 0.05; *p* < 0.01; and *p* < 0.001. All statistical analyses were conducted using GraphPad Prism version 6 (GraphPad, La Jolla, CA, USA).

## Figures and Tables

**Table 1:** Structure and activity of hydrazine-based pyrrolidine-2-one (**2a**–**g**) and related compounds (**3**–**6**).

Entry	Compound	Linker	R	M (g/mol)	MIC (µg/mL)
1	**2a** (Hyd.H)	-NH-NH-	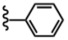	191.23	9.6
2	**2b** (Hyd.OCH_3_)	-NH-NH-	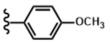	221.26	11.1
3	**2c** (Hyd.Cl)	-NH-NH-	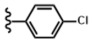	225.68	5.6
4	**2d**	-NH-NH-	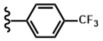	259.23	130
5	**2e**	-NH-NH-	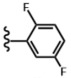	227.21	114
6	**2f**	-NH-NH-	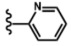	192.22	96.1
7	**2g**	-NH-NH-	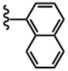	241.29	121
8	**3**	-NH-	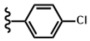	210.66	105
9	**4**	-NH-CH_2_-		224.69	112
10	**5**	-NH-NH-CO-		253.68	127
11	**6**	-CO-NH-N=CH-		265.70	133

**Table 2:** Description of the *C. albicans* strains used in the current study and their MICs.

Strain	Description	Caspofungin MIC (µg/mL)	Fluconazole MIC (µg/mL)	Ref.
*C. albicans* SC5314	Wild-type	0.03	0.5	[12]
*C. albicans* 15351c6859	Venous catheter, caspofungin-resistant	4	1	[13]
*C. albicans* 15343c3523	Blood, caspofungin-resistant	2	0.5	[13]
*C. albicans* 17287c305	Blood, caspofungin-resistant	8	0.5	[13]
*C. albicans* 92535989	Tracheal secretion, fluconazole-resistant	0.06	64	[13]
*C. albicans* 14316c1746	Bronchoalveolar lavage, fluconazole-resistant	0.03	128	[13]
*C. albicans* 14294c5335	Stools, fluconazole-resistant	0.06	5	[13]

## Data Availability

The data presented in this study are available in the article.
